# Post-Transplant Ureteric Stricture Managed By Extra-Anatomical Stenting With Modification on the Originally Described Stent Location: A Case Report

**DOI:** 10.7759/cureus.33335

**Published:** 2023-01-04

**Authors:** Omar Buksh, Ahmed Khogeer, Abdullah O Bawazir, Adel Alammari, Islam Junaid

**Affiliations:** 1 Urology, King Faisal Specialist Hospital and Research Center, Jeddah, SAU; 2 College of Medicine, King Saud Bin Abdulaziz University for Health Sciences, Jeddah, SAU

**Keywords:** ureteric stricture, extra anatomical stent, ureteral stents, kidney transplant ureteral stricture, ureteral stricture management

## Abstract

Ureteric strictures are a relatively uncommon complication following renal transplant, which may be managed endoscopically or surgically by repairing the stricture. Extra-anatomical bypass is a useful procedure in complex cases that bypasses the ureter by creating a subcutaneous route, although it is uncommonly used given its rare indication. We report a case of renal transplant ureteric stricture, in which we utilized a modified extra-anatomical stenting technique with a Detour® stent to avoid the fibrotic planes surrounding the lateral aspect of the kidney graft.

## Introduction

A kidney transplant improves survival rates and quality of life in patients with end-stage chronic renal failure compared to dialysis [1].In the past few decades, kidney transplant outcomes have improved noticeably, with operative complications dropping from 30% to 10% [2]. However, ureteral stricture post-renal transplantation can lead to failure or dysfunction of the transplanted kidney graft [3]. Moreover, ureteral stricture at the ureterovesical anastomosis is the most common post-transplantation complication with a reported occurrence of 0.5% to 10% [4,5]. These ureteral strictures are managed conservatively by decompression using a retrograde stent or a percutaneous nephrostomy tube, but they still have a recurrence rate of 45% [3]. The most effective management is by open surgery of ureteral anastomosis or reimplantation, which yields favorable long-term outcomes [1,6].

Extra-anatomical stenting (EAS) for transplant ureteric strictures is a rarely used surgical option when endoscopic or open reimplantation or reconstruction is not feasible [7]. We present a slight modification to the originally described technique by tunnelling the Detour® (Coloplast, Humlebaek, Denmark) medially into the transplanted kidney with a complex transplant ureteric stricture.

## Case presentation

**Figure 1:**
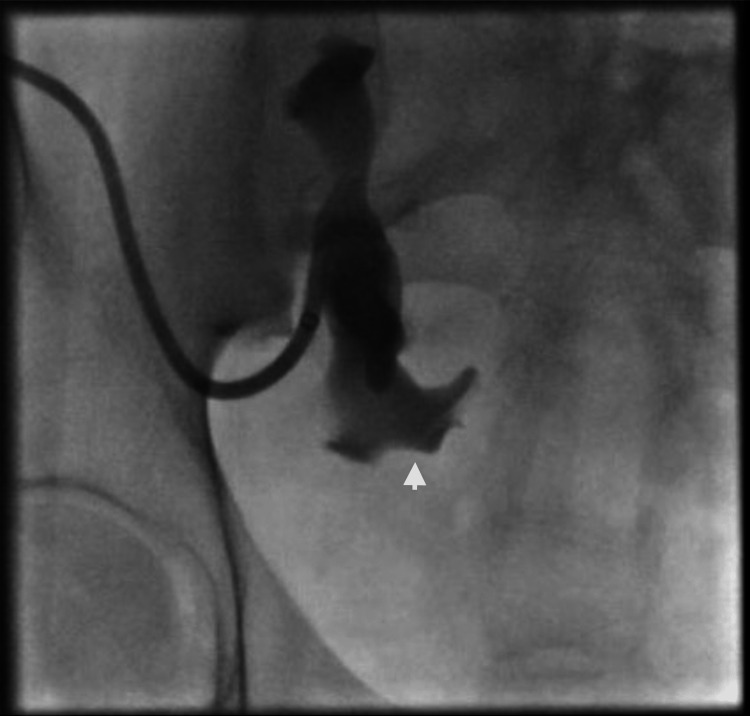
Nephrostogram showing complete obstruction at the level of the renal pelvis (indicated by the arrow) with no contrast passing to the ureter.

The patient is a 28-year-old male with a history of epilepsy and hypertension. He underwent a living unrelated renal transplant (LURRTx) 2 years ago and was receiving oral prednisolone, mycophenolic acid, and tacrolimus. The early postoperative course was complicated by transplant ureteric stenosis that resulted in acute kidney injury (AKI), hydronephrosis, and complete obstruction of the transplanted ureter. The nephrostogram images revealed fibrosis of the entire transplanted ureter (Figure 1). An attempt at open ureteric stricture repair was unsuccessful and complicated by intra-operative bleeding and difficulty in identifying the ureter.

At presentation in our outpatient clinic, the patient was vitally stable and afebrile and had normal general examination. His abdominal examination was significant for scars on the right lower abdomen. Nephrostomy tube was draining turbid urine, with a baseline serum creatinine between 120 and 140 Umol/L, the rest of blood workup was unremarkable, and urine culture from nephrostomy tube was positive for *Pseudomonas aeruginosa*.

The patient received intravenous antibiotic (IV) treatment and nephrostomy tube exchanges to clear the *Pseudomonas aeruginosa* infection. Nephrostogram (Figure 1) showed complete occlusion at the level of the renal pelvis and no contrast passing to the ureter. Antegrade stenting in the past had failed due to complete ureteric occlusion. An attempt to perform an open reconstruction was also unsuccessful due to dense scarring and bleeding.

Extra-anatomical ureteric stenting using Detour was considered the next most appropriate option to drain the transplanted kidney. The patient was counseled and consented to the procedure.

In preparation for the Detour operation, the patient was admitted, and new urine cultures were sent from the nephrostomy site, which showed *Staphylococcus aureus*. The patient received an extended course of IV cefazolin as per the recommendation of our Infectious Disease Department. A new nephrostomy tube was inserted at a different tract in order to avoid the previous nephrostomy site.

**Figure 2:**
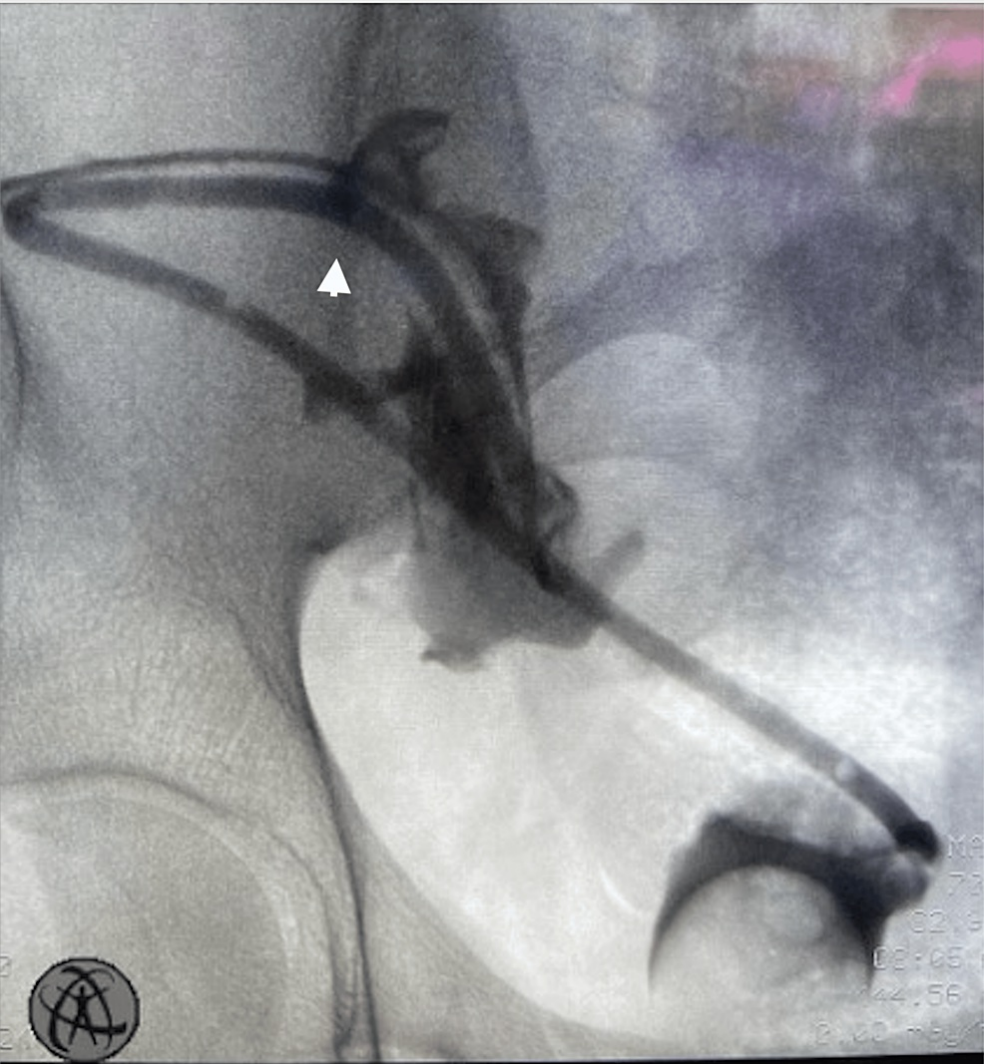
Nephrostogram performed intraoperatively showing the contrast delineating the extra-anatomical stent and filling the bladder. The arrow marks the approximate location of the exit from the kidney.

**Figure 3:**
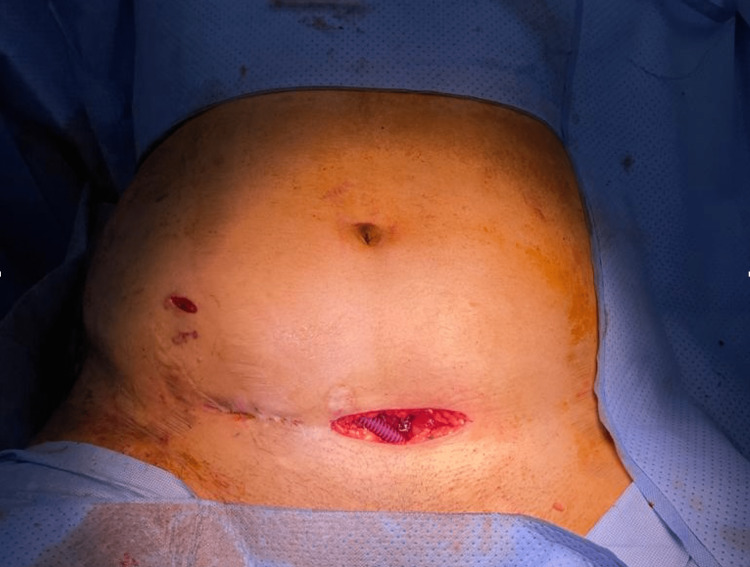
Prior to skin closure, the Detour stent was placed medially to avoid the scars and fibrotic tissues surrounding the renal graft.

The patient underwent the procedure after confirming no growth on urine culture. Under general anesthesia, the procedure started with a cystoscopy, which showed a mildly trabeculated bladder. The bladder was then accessed and dissected via a low transverse incision 2 cm above the symphysis pubis to receive the distal end of the Detour stent. The nephrostomy tube was removed over a guide wire, and the tract was dilated with difficulty using a balloon dilator due to the significant fibrosis surrounding the renal capsule. The proximal end of the Detour was positioned within the collecting system of the transplanted kidney under fluoroscopic control. The Detour tunnelling device was used to position the Detour stent from the nephrostomy site medial to the transplanted kidney to avoid the significant fibrotic tissue surrounding the graft. The distal end was delivered to the bladder (Figure 2). The length of the stent was adjusted by cutting away the excess length. Cystotomy was performed between stay sutures and the denuded silicone tube was introduced into the bladder. Cystotomy was closed securely around the stent anchoring using the outer PTFE (polytetrafluoroethylene) tube of the Detour using interrupted polydioxanone sutures (Figure 3).

The patient was then placed on a Foley catheter to drain the bladder for one week. Post-operative course was uneventful, and the patient was discharged home with a creatinine level of 110 umol/L and an estimated glomerular filtration rate (eGFR) of 40 mL/min. At 12 months’ follow-up, the patient remains well with an eGFR of around 40 mL/min and remains infection free.

## Discussion

While complex ureteral strictures can be treated with a variety of surgical options, several factors contribute to their success and should be considered in proper planning and management [8]. For instance, the treatment of ureteral stricture in patients with declining renal function and symptoms is traditionally decompression using a nephrostomy tube or stent, followed by a surgical repair [9]. When decompression stents fail in treating the stricture, open or laparoscopic interventions (e.g., Boari flap, ureteroureterostomy to the native ureter, or vesicopyelostomy) are required [10].

EAS or nephrological stenting involves the creation of a subcutaneous tunnel through the renal pelvis to bypass the stricture ureter [11]. Initially applied to native kidneys in the 1960s, EAS has shown promising long-term results in both benign and malignant conditions [12]. EAS can be used either temporarily or permanently. Due to encrustation and stent blockage, a temporary stent requires replacement after 6 to 12 months. However, a permanent stent "Detour" does not require exchange and provides a long-term solution [13].

Detour is a self-retaining coaxial tube. The outer tube is PTFE, and the inner silicone tube is reinforced by plastic rings. The proximal end of the stent is marked with a radio-opaque marker between the inner and outer PTFE layers. The kit includes a 30Fr Amplatz sheath and a large subcutaneous tunneling device [14,15].

Extra-anatomic bypass has a high patency rate of 90.9%, with no intraoperative complications. Stent infection was the most common long-term complication with a 38.5% rate, but the infection usually occurs after four years of implantation [8]. In complex kidney ureteric strictures, EAS displayed stable renal function for 32 months [14,15]. Using EAS with a regular exchange on a patient with renal transplant stricture, Tahir et al. reported that the renal graft function remained intact after 18 months and without complications [12]. Furthermore, EAS is useful in complex ureteric strictures that have failed antegrade and retrograde stenting in patients who do not desire a long-term nephrostomy tube or malignant conditions [11].

The EAS offers several advantages over both percutaneous and posteriorly positioned nephrostomies, including better hygiene and psychosocial outcomes [11,12], although EAS is associated with complications including urinary leakage, urinary tract infection, stent dislodgement and kinking, and prolonged vomiting. However, these complications can be prevented by exchanging the stent yearly [11,12].

As our patient had a significant previous history of failed open repair of the transplant stricture and multiple renal graft pyelonephritis, we considered placing the EAS Detour medially to avoid the dense fibrotic tissue surrounding the graft, which led to normal graft function with an uneventful course. The technical modification of placing the Detour medially, instead of laterally, as originally described, serves as an adequate alternative when avoiding the fibrotic tissues around the renal graft is necessary [16,17].

In regard to quality of life, EAS is advantageous in cases with long life expectancy that have exhausted other options of treatment, as EAS avoids the psychological burden of an indwelling nephrostomy tube or the discomfort associated with ureteral stents and the necessity of exchanging them regularly [18].

## Conclusions

Ureteral stricture is a challenging urological complication post-renal transplantation. EAS is a useful option for certain cases that are difficult to manage surgically and is associated with good quality of life and renal function outcomes. Placing the EAS medially to avoid the fibrotic tissues surrounding the renal graft serves as a good alternative. As the use of such devices increases, more data will be available to expand on the available safety and efficacy data.
